# A new vessel filled and heart-beating human corpse model for VATS lobectomy training

**DOI:** 10.1007/s00464-024-11119-9

**Published:** 2024-08-19

**Authors:** Vincenzo Verzeletti, Luigi Lione, Alessandro Bonis, Nicolò Sella, Giorgio Cannone, Luca Melan, Alessandro Rebusso, Eleonora Faccioli, Andrea Porzionato, Giovanni Maria Comacchio, Samuele Nicotra, Andrea Dell’Amore, Federico Rea

**Affiliations:** 1 Thoracic Surgery Unit, Department of Cardiac, Thoracic, Vascular Sciences and Public Health, University of Padua, Padua, Italy; 2 Institute of Anesthesia and Intensive Care, Padua University Hospital, Padua, Italy; 3 Department of Neurosciences, Institute of Human Anatomy, University of Padua, Padua, Italy

**Keywords:** Cadaver model, Beating heart, VATS training, VATS lobectomy, Education, Minimally invasive training

## Abstract

**Background:**

Nowadays, video-assisted thoracic surgery (VATS) lobectomy represents the treatment of choice for early-stage lung cancer. Over the years, different methods for VATS training have evolved. The aim of this study is to present an innovative beating-heart filled-vessel cadaveric model to simulate VATS lobectomies.

**Methods:**

Via selective cannulation of the cadaver heart, the pulmonary vessels were filled with a gel to improve their haptic feedback. An endotracheal tube with a balloon on its tip then allowed movement of the heart chambers, transmitting a minimum of flow to the pulmonary vessels. A simulated OR was created, using all instrumentation normally available during surgery on living patients, with trainees constantly mentored by experienced surgeons. At the end of each simulation, the participants were asked 5 questions on a scale of 1 to 10 to evaluate the effectiveness of the training method (“1” being ineffective and “10” being highly effective).

**Results:**

Eight models were set up, each with a median time of 108 min and a cost of €1500. Overall, 50 surgeons were involved, of which 39 (78%) were consultants and 11 (22%) were residents (PGY 3–5). The median scores for the 5 questions were 8.5 (Q1; IQR_1–3_ 8-9), 8 (Q2; IQR_1–3_ 7–9), 9 (Q3; IQR_1–3_ 8–10), 9 (Q4; IQR_1–3_ 8–10), and 9 (Q5; IQR_1–3_ 8–10). Overall, the model was most appreciated by young trainees even though positive responses were also provided by senior surgeons.

**Conclusions:**

We introduce a new beating-heart filled-vessel cadaveric model to simulate VATS lobectomies. From this initial experience, the model is cost effective, smooth to develop, and realistic for VATS simulation.

**Supplementary Information:**

The online version contains supplementary material available at 10.1007/s00464-024-11119-9.

Nowadays, early-stage lung cancer is predominantly addressed through minimally invasive surgical approaches [1, 2]. The adoption of these techniques has inevitably altered surgical practices, affecting senior surgeons who are unfamiliar with video-assisted procedures, younger surgeons who are still not completely skilled in video-assisted thoracic surgery (VATS), and residents who are facing challenges in acquiring adequate training, mainly due to the lack of a teaching model [2, 3]. This steep learning curve is a substantial limitation to the development of surgical skills, considering that surgeons in training need time to learn all steps necessary to safely perform minimally invasive surgery [4, 5]. Therefore, considering that proficiency in a VATS lobectomy is only acquired after 25–50 cases (or even over 200 cases without intensive mentorship), it's crucial to have educational tools that can keep up with swift surgical evolution [6]. For this purpose, different training models have been developed over the years: human cadavers and live animals for realistic practice, wet and dry trainer boxes with animal tissues or synthetic models for procedure simulation, and virtual/augmented reality simulators for immersive and complex-scenario training [7]. Despite these innovations, the prevailing VATS lobectomy training model often still follows the traditional OR-based concept of “one teach, one observe” [7].

In our center, we have gradually introduced surgical simulation on human corpses alongside conventional OR-based training [8, 9]. Cadaveric simulation provides enhanced fidelity in replicating real surgical environments, including patient positioning and the execution of complex surgical maneuvers [8, 9]. This provides a more comprehensive and realistic experience in comparison to other training methods, such as wet or dry boxes or virtual simulations, which often fall short in emulating an intricate and realistic surgical setting [6, 10].

The aim of this paper is to present an innovative beating-heart and filled-vessel cadaveric model. This was tested for the simulation of VATS lobectomies as well as to explore the personal opinions of those surgeons involved in this trainee program, allowing us to constantly improve and eventually perfect this new training model.

## Methods

### Human corpse

All the procedures in this study were performed on fresh-frozen human bodies provided by the in-house corpus donation program “Donation to Science” from the Veneto Region Reference Center for the Preservation and use of Gifted Bodies (DGR Veneto Region n. 245, 8th March 2019; N389897) [11]. All the corpses were intact, stored at −20 °C and had no history of previous chest surgery or trauma. 96 to 120 h before dissection the corpses were defrosted and brought to the dissection room.

### The new VATS training model

**Fig. 1:**
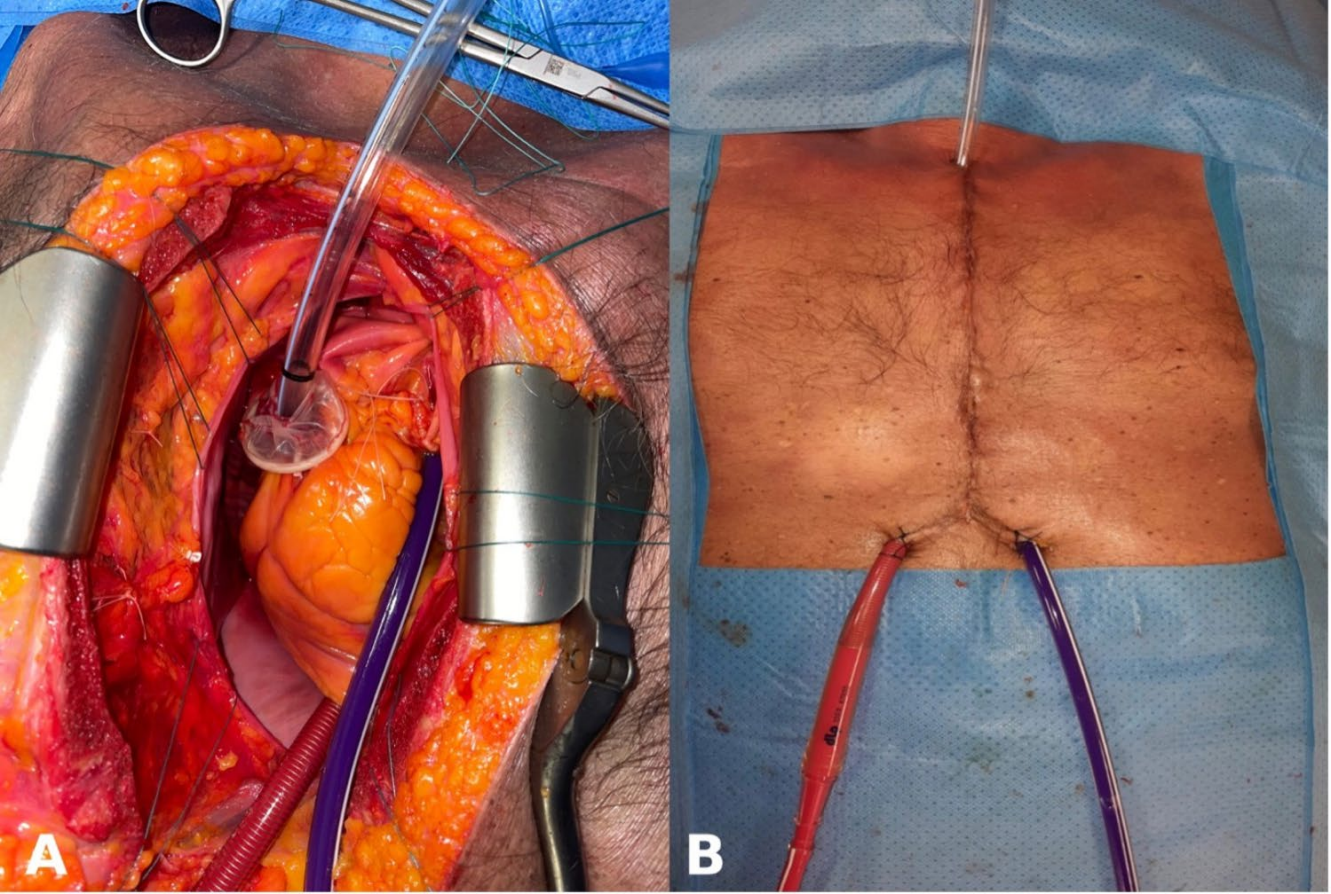
The picture (**A**) depicts the appearance of the model following the placement of the endotracheal tube into the right ventricle and the two perfusion cannulas in the main pulmonary artery (blue) and in the left atrium (red). Panel **B** shows the model at the end of the preparation and ready for surgical simulation

**Fig. 2:**
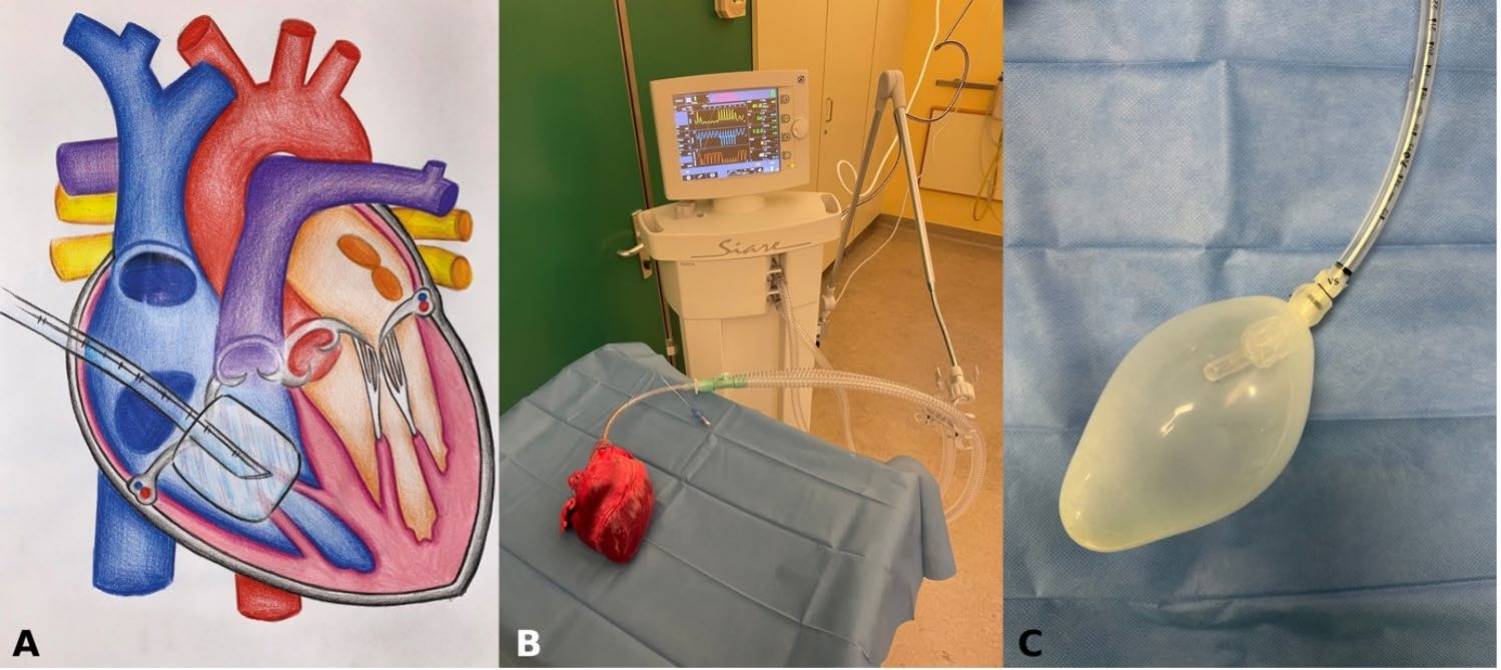
The picture (**A**) shows the endotracheal tube inserted into the right ventricle through a small atriotomy, passing through the tricuspid valve. Panel **B** shows the isolated heart-beat simulation system in a swine heart. Panel **C** shows in detail the inflated balloon at the end of the endotracheal tube

We have slightly modified and improved the previous model for VATS lobectomy training and simulation on human corpses [8], including a dynamic system that simulates cardiac pulsatility and allows a minimal transmitted flow to pulmonary circulation (Figs. 1, 2).

This dynamic system uses a non-stiff cannula, which is directly inserted into the pulmonary artery (PA) just above the pulmonary valve plane, while a straight cannula (usually employed for inferior vena cava cannulation) is placed in the left auricle (Fig. 1).

Following this, the lower end of a 7.5 mm endotracheal tube is connected to an ultrasound probe cover, which is able to form a balloon on the end of the tube. This is then inserted into the right ventricle through a small right atriotomy, passing through the tricuspid valve (Fig. 2). The tube is connected to a mechanical ventilator that inflates and deflates the balloon, mimicking the motility of the ventricle (Fig. 2**, **Video 1). The ventilator is set in pressure-controlled mode: inspiratory pressure and positive end-expiratory pressure were previously titrated on an isolated pig heart on the basis of balloon/heart compliance (Fig. 2), in order to deliver a tidal volume of approximately 150 mL (i.e., 30 cmH_2_O inspiratory pressure) and simulate an end-systolic volume of approximately 50 mL (i.e., 8 cmH_2_O positive end-expiratory pressure). At the same time, the respiratory rate was set at 60 to 80 breaths per minute in order to simulate a normal human heart rate. The movement of the cardiac chambers not only simulates the heartbeat, but also gives a transmitted flow to the pulmonary circulation (Video 2).

After this, the PA and the left atrium perfusion cannulas are connected to two separate 3/8 tubes for extracorporeal circulation and the system is filled with a colored gel, as previously described [8]. Initially the static perfusion solution is infused under vision to exclude gel leakage and to ensure correct filling of the intrapericardial pulmonary vessels [8], after which the pericardium is sutured and the chest is closed (Fig. 1).

Finally, a single lumen endotracheal tube is placed into the trachea via the mouth and connected to a breathing bag balloon, which allows the ventilation of the lung during the surgical simulation. The corpses are set up the day before the surgical simulation and are left in the simulation room overnight until the next morning.

### VATS lobectomy simulation

**Fig. 3:**
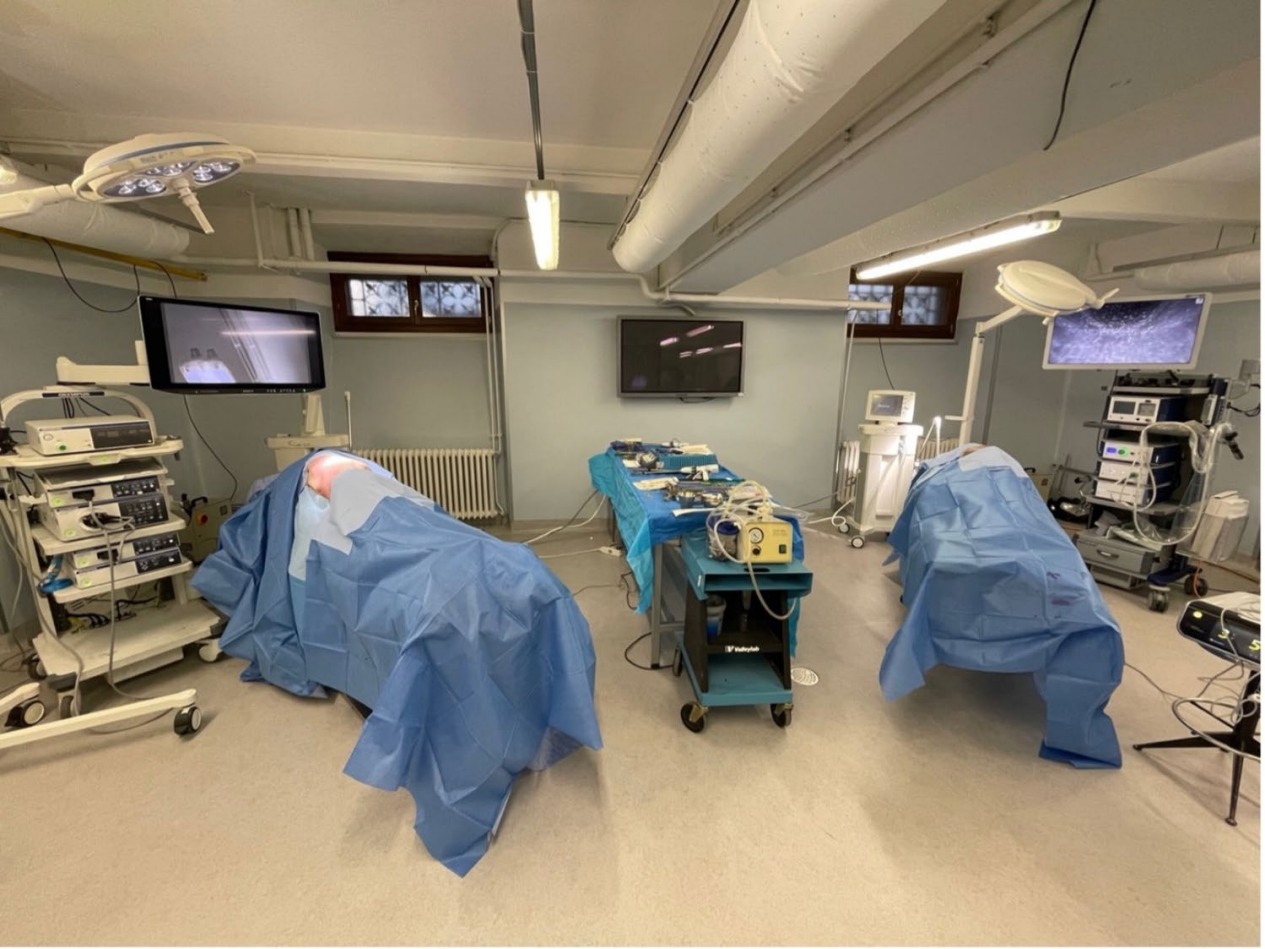
Setting of the surgical simulation with corpses positioned in lateral decubitus

On the day of the hands-on course, the corpse is placed on a lateral decubitus, according to the side of the lobectomy, and the surgical field is draped in the same way as in clinical practice (Fig. 3). The ends of the two conduits, each filled with a gelatinous substance and securely clamped, are positioned externally to the surgical area, near the cadaver’s pedal extremities. Before beginning the simulation, the systems are further filled with the colored gelatinous substance. This process continues until significant resistance to the influx is clearly detected, after which the conduits are resecured with clamps to avoid backflow. VATS lobectomies are performed using a 3-port anterior approach. The first incision is made in the fourth intercostal space, while the other 2-ports at the level of the seventh intercostal space on the median axillary and the scapular tip lines. The lobectomy is then performed with the same technique we use in the OR. Trainees go through the different steps of the surgical procedure under the supervision of experienced faculty members and with the assistance of an expert scrub nurse (Video 2). The surgical instruments are the same as those used in clinical practice for minimally invasive surgery and the camera for all procedures is a 3D-vision model (Fig. 3).

### Evaluation and statistics

At the end of all the surgical simulations, the trainees were asked to answer the following questions, ranking their answers between 1 and 10 (“1” indicating lower effectiveness/satisfaction, and “10” indicating high levels of satisfaction):

1. How realistic was the model set-up? (Question 1, Q1)

2. How comparable was the quality of the cadaver tissue to that of living patients? (Question 2, Q2)

3. How comparable was the handling of the surgical instruments to that in clinical practice? (Question 3, Q3)

4. How faithful were the surgical steps applied to the model compared with those applied on a living patient? (Question 4, Q4)

5. What was your overall degree of satisfaction in performing the simulation? (Question 5, Q5)

For each VATS lobectomy, the supervisors reported whether all surgical steps were completed correctly and if any complications occurred.

The study participants were categorized according to:

– Previous VATS experience as lead surgeon: Group A had performed < 30 VATS lobectomies, Group B > 50 VATS lobectomies, and Group C between 30 and 50 VATS lobectomies.

– Surgical training status (i.e., if they were consultants who already completed their formal surgical training, or residents who were still in training).

### Statistical analysis

Continuous variables were reported as median and interquartile range (IQR) while categorical variables were registered as number and percentage. Comparisons were performed using the Wilcoxon test or the Kruksal–Wallis test when appropriate.

Plots were obtained using R statistical software (tidyverse package), while comparisons were obtained with Jamovi software.

## Results

### Analyses of our preliminary experience

Eight models were set up with a median time of 108 min (IQR_1–3_ 105–121) required for corpse preparation.

We performed four hands-on sessions using two human cadavers for each session. The cost of setting up a single model was about €1500. This estimate takes into account the purchase of a disposable bronchoscope, as well as costs related to surgical instrument sterilization, rental of the endoscopic system and camera on the simulation day, ultrasonic scalpel rental, plus costs related to surgical drapes, and cannulas for cardiac cannulation. Mechanical staplers were obtained from stockpiles, so did not incur any costs. The corpse was also not purchased since it belonged to our in-house donation program. For each cadaver, four lobectomies (right upper, right lower, left upper, and left lower) were performed.

Overall, 50 surgeons were involved, 39 (78%) of which were fully trained (consultants) and 11 (22%) were residents (Post-Graduation Year 3–5). Among the study participants, 6 (12%) had previously performed more than 50 VATS lobectomies as head surgeon (Group B; none of which were residents), 14 (28%) had performed 30–50 VATS lobectomies (Group C; 1 was a resident) and 30 (60%) had performed less than 30 procedures (Group A; 10 of which were residents). From Group A, 18 (36%) surgeons had performed 10–30 VATS lobectomies (1 was a resident) and 12 (24%) had performed less than 10 VATS lobectomies as head surgeon (9 of which were residents).

During the simulation session, each surgeon performed a single VATS lobectomy as head surgeon or at least one lobectomy as an assistant. All surgeons were able to complete all the steps of the operation. General information on the answers given to our questionnaire is reported in Table 1.

**Table 1:** Results of questionnaire answers for all participants (overall) and for participants subdivided by group (A, B, and C)

Question	Overall (*N* = 50)	A (*N* = 30)	B (*N* = 6)	C (*N* = 14)	*p*-value
Q1	8.5 (8–9)	9 (8–10)	8 (7–8)	8.0 (7–9.1)	0.02
Q2	8 (7–9)	8 (7–8.1)	7.5 (6.9–9)	8.5 (7–9)	0.75
Q3	9 (8–10)	9 (8–10)	8 (8–8.1)	9 (8–10)	0.12
Q4	9 (8–10)	9 (8–10)	8 (7.9–8.1)	9 (8–10)	0.06
Q5	9 (8–10)	10 (8–10)	8.5 (7.9–9.1)	9 (8–10)	0.11
Total score	43 (41–45.8)	43.5 (41–46)	40.5 (38.7–41.2)	45 (39.9–46.1)	0.07

Data are median (IQR)

### Comparisons between surgeons with difference experience

**Fig. 4:**
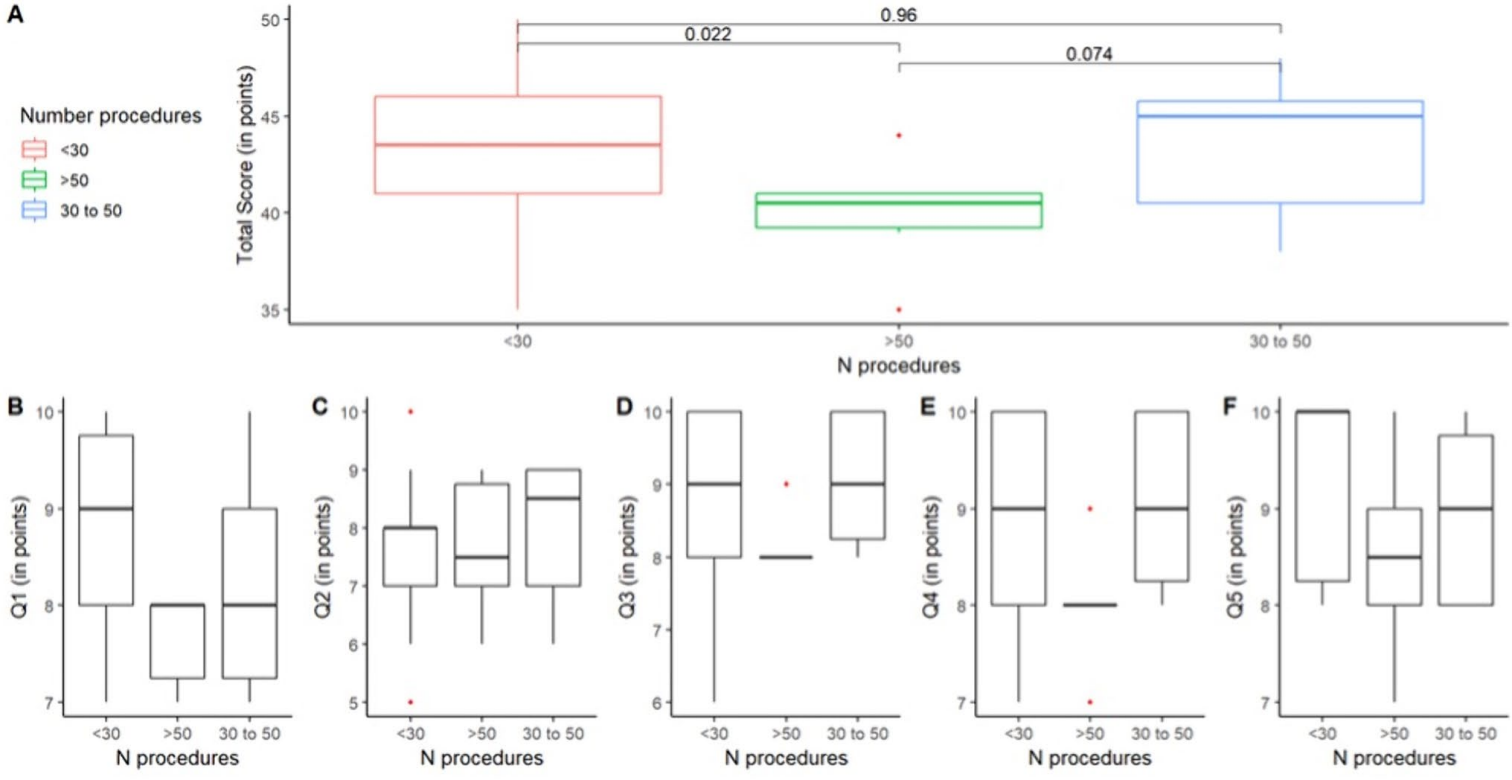
Comparisons among groups according to their surgical experience. In (**A**) is reported comparison concerning the total score of the questionnaire. In (**B**) to (**F**) are reported comparisons divided question by question (Qn)

Differences in questionnaire scores according to previous VATS experience are shown in Fig. 4. In particular, participants with more than 50 previous VATS lobectomies (Group B) reported a median total score of 40.5 points (IQR 38.7–41.2), which was significantly lower than that reported by participants with less than 30 previous VATS lobectomies (Group A) (with a median of 43.5 points; IQR 41–46, *p* = 0.022). When comparing Group B to the participants with 30 to 50 previous VATS lobectomies (Group C), there is no statistical difference in scores (median 45.0 points; IQR 39.9–46.1, *p* = 0.074). The difference between Group A and C was also not significant (*p* = 0.96). When answering Q1, Group A provided a median one-point higher score than the other groups (*p* = 0.026). Q2 led to a general agreement between the three groups (*p* = 0.750), with Group A giving a median score of 8.0 (IQR 7.0–8.1), Group B a median of 7.5 (IQR 6.9–9), and Group C giving 8.5 (IQR 7–9). For Q3 and Q4, Groups A and C answered similarly (median score of 9, IQRs 8–10 for both questions). A significant difference was found between Group B and Groups A and C when looking at their answers to Q1 (*p* = 0.02) and Q4 (*p* = 0.06) (Table 1).

### Perspective based on experience level

**Fig. 5:**
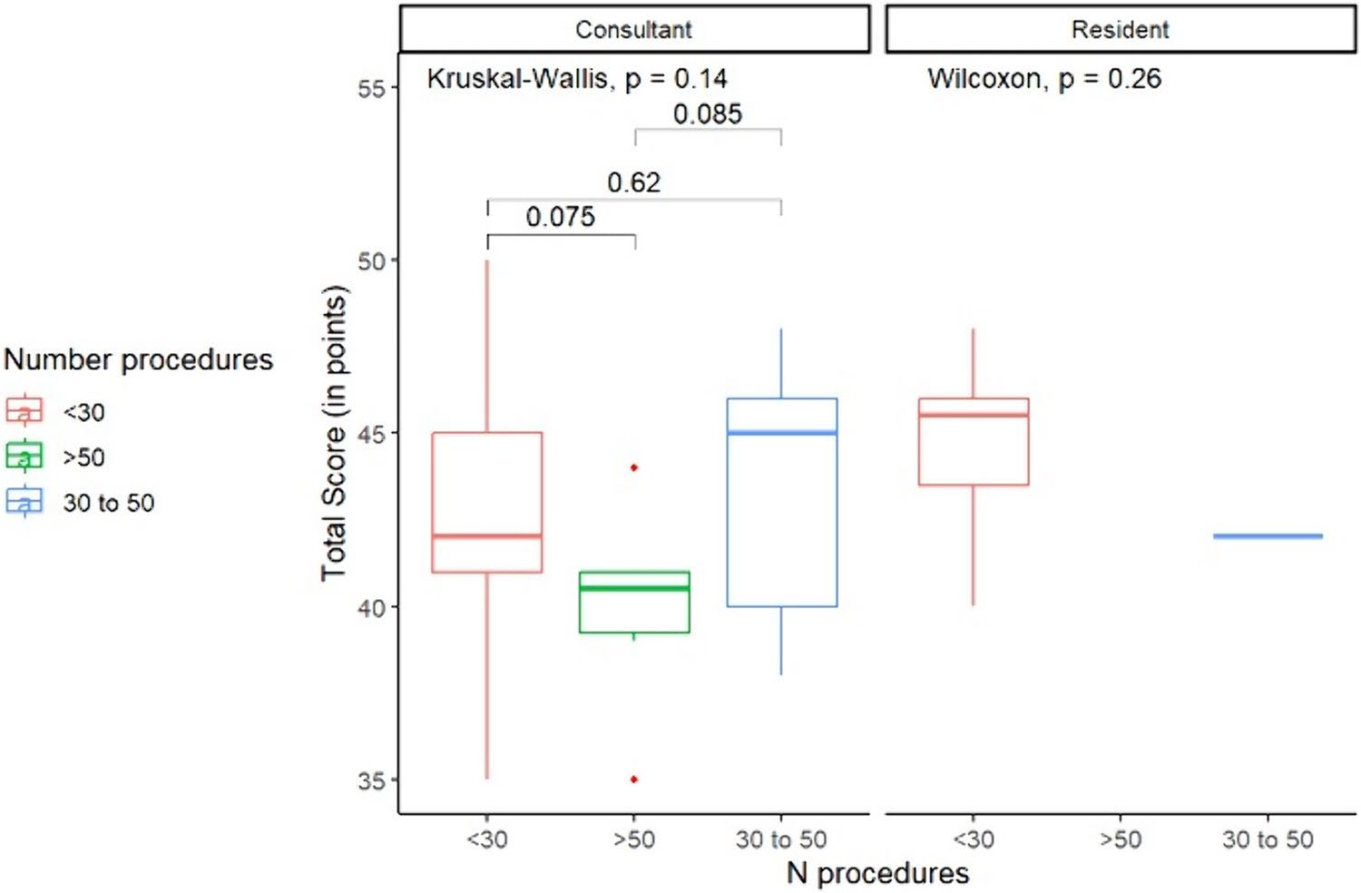
Comparisons concerning the total score of the questionnaire between residents and consultants and subdividing each group for number of procedures

Differences in the score between consultants (fully trained surgeons) and residents (in-training) are reported in Table 2 and Fig. 5.

**Table 2:** Results of questionnaire answers for participants subdivided by experience level (consultants or residents)

Question	Consultants (*N*=39)	Residents (*N*=11)	*p*-value
Q1	8 (8–9)	10 (9–10)	<0.01
Q2	8 (7–9)	8 (7–8)	0.30
Q3	9 (8–10)	9 (8.5–10)	0.17
Q4	9 (8–10)	9 (9–9.5)	0.23
Q5	9 (8–10)	10 (9–10)	0.14
Total score	42 (40–45)	45 (43–46)	0.07

Data are median (IQR)

Overall, a non-significant trend to a median lower total score was given by consultants (42.0 points, IQR 40.0–45.0) while residents seemed to be more satisfied after their experience (45.0 points, IQR 43.0–46.0) according to the Wilcoxon rank sum test (*p* = 0.07).

According to the comparative analysis, Question 1 and Question 5 highlighted the main differences between the experiences of the residents and the trained consultants. Q1 is related to how realistic the design felt, both in terms of the environment and the surgical set-up. Trained consultants gave a lower median score for Q1 compared to residents (*p* < 0.01), with a median of two points difference. The second main aspect that differed between consultants and residents regarded the overall effect given by the cadaver surgical lab. Despite having no statistical significance, Q5 highlighted that the residents considered this cadaver-assisted VATS surgery to be an improvement on the current system, while trained surgeons gave a median of one point less (*p* = 0.14). Residents and consultants were generally in agreement on Q2 (*p* = 0.3), Q3 (*p* = 0.17) and Q4 (*p* = 0.23) (Table 2), concluding that the cadavers had a generally acceptable tissue quality in comparison with living patients.

## Discussion

### General considerations on human corpse surgical training

Our experience regarding cadaver-based surgical training is reported in this paper.

The creation of this model for minimally invasive surgery simulation represents the culmination of our work on human corpse-based training, the details of which can be found in our reports of simpler past models [8, 9]. In our opinion, when complex models are employed (such as the one presented herein), cadaver surgical training ranks among the most promising techniques available for surgical simulation. In addition to the faithful representation of anatomy and realistic quality of tissue handling that cadaver training provides, adding in a simulated heartbeat reproduces part of the dynamic scenario experienced in live patients, and thus overcomes one of the major limitations of human corpse models. This is also one of the major strengths of surgical training on live anesthetized animals, such as pig models [12, 13]. These models provide a training platform that offers satisfactory anatomical fidelity, realistic tissue manipulation, and the capability to simulate critical incidents (such as a hemorrhage) within a clinically safe environment [12]. However, they do pose considerable ethical problems related to the sacrifice of animals for surgical training, which is precisely the reason why such models are being used less and less frequently [8]. It also goes without saying that the interspecies differences create an unbridgeable gap between training and reality when using these live animal models.

Beyond the general advantages already explained above and in a previous report [8], this new dynamic model is unique as it maximizes surgical simulation via the heartbeat and transmitted flow to the pulmonary circulation (Video 1). Both trainees and senior surgeons considered the motility of the cardiac chambers in the VATS setting to be fairly realistic and appreciated the turgidity and the simulated flow within the vessels. All of this is provided by our model with a fairly quick set-up that is easy to reproduce, and which has a rather moderate cost when compared to other models [14]. Over the years, some complex models have been published for cardiac and vascular surgery training in which the circulation was simulated at paraphysiological pressures via the use of perfusion pumps [15–17]. While impressive, this does incur comparatively higher costs. All in all, we consider our model to be a satisfactory solution for VATS lobectomy training at a moderate cost and with reasonable preparation, except for cases in which the specific simulation of vascular complications is necessary.

Naturally, cadaveric models also present some drawbacks that should be mentioned. First of all, a cadaver-equipped facility for surgical training involves significant costs associated with cadaver preservation, facility maintenance, and related expenditure. Availability of cadavers also varies depending on geographic location, ethical and religious considerations, and institutional resources, thus potentially limiting access for surgical training, particularly in underprivileged regions [11]. In house-corpus donation programs are not yet widespread and thus paid-for corpses are often used for surgical training, again with not insignificant costs [11].

Moving on from the main aims of our study, this corpse model is also worthy of two other points of discussion. First, these cadaver models were not discarded after just five lobectomies. After completing our survey (to avoid any risk of bias), other procedures were also performed by residents (i.e., thoracic wall resections and reconstructions, mediastinoscopy, complex thoracotomies, tracheostomy) under the supervision of the residency program’s tutors. Thus, residents had the chance to perform difficult and unusual procedures that they otherwise would rarely have had the chance to practice. This brings us to our second point, namely that this corpse model is a multi-course human model. This means that it is possible to use the same corpse for different training sessions in different surgical specialties, leading to a reduction in overall costs.

### Further considerations and questionnaire interpretation

Strictly considering our cohort, a couple of criticisms need to be addressed: first of all, the modest number in the cohort leads to cautious statistical interpretations when drawing conclusions. Secondly, the questionnaire is focused on the subjective experience of cadaver training, which of course presents a challenge in objectivity, despite the scoring system that we developed.

### Considering these two caveats, our study did uncover some interesting insights

It is interesting to note that Group A (which had the least experience) gave a median score that was 1.5 points higher than Group B (the most experienced surgeons) for Q5, reflecting the greater benefit that surgeons at the beginning of their learning journey felt when approaching a VATS lobectomy through cadaver-assisted training. This is also confirmed when analyzing the total score (Fig. 4), where Group A provided a median higher score in comparison with Group B.

In general, we could say that the lower the number of previous procedures, the higher the total score, suggesting that greater satisfaction was achieved with individuals that had performed fewer surgical procedures as lead surgeon.

This led us to further investigate whether these scores could be determined by (a) the number of operations performed as lead surgeon or (b) their qualification (resident or consultant). While there was no significant difference in total score, both Q1 and Q5 were the most disputed questions, underlying that the environmental set-up and the overall satisfaction during the training had variability between the three groups. Most likely, the more skilled surgeons had higher expectations in terms of realism, which is naturally difficult to obtain, and probably could also discern variability in the conservation status of the bodies used. Another explanation for the trained surgeons having lower satisfaction could be related to their learning curves. Indeed, it is fair to assume that they have already acquired the minimal skills needed to perform a safe VATS lobectomy, which is one of the major aims of this traineeship. It could thus be more interesting for this group to use the same model to perform more complex VATS procedures, such as sleeve lobectomies or segmentectomies.

Following this logic, residents may have given a higher score because of their limited experience as a VATS lobectomy lead surgeon. Undoubtedly, this group had a lower need for extreme realism, and instead may have merely appreciated the chance to improve their surgical skills in a context that resembled the OR, but also guaranteed a safe environment.

Group C (the intermediate-skilled surgeons, with 30–50 procedures) provided the highest total score, suggesting that they particularly appreciated this training model. As this group already had some solid training in VATS lobectomies, they may have appreciated the realism of our model, which allowed them to boost their skills in specific steps of the procedure, which could then be applied to their everyday practice.

Overall Group A (<30 VATS lobectomies) seemed to be less satisfied than the intermediate group (Group C) by this experience. This could be explained by a difference in expectations between the two groups. While the latter have already conquered the steepest part of the learning curve (about 20 lobectomies) and are looking for more refinement, Group A may instead be looking for fast improvement. Perhaps then other models could better fulfill their expectations. For instance, an augmented reality training course would allow more procedures to be performed in a single same day (both in terms of number and type). This is a substantial limitation of the corpse model, as it has a limited number of performable procedures (five lobectomies per corpse). Furthermore, corpse models are not available everywhere, and in some cases consultants with lower surgical experience could be less interested, due to difficulties in reproducing this experience outside our training session. This leads us to one of the main limitations of this new model, which is the cost of purchasing the corpse, as other costs would be the same for an animal model. This is why we believe that our model can find its greatest application in centers that have an in-house donation program.

To conclude, on the one hand this new beating-heart VATS surgery model is worthy of further investigation, considering its adherence to real-life intraoperative VATS scenarios. On the other hand, based on our results, it is clear that one model doesn’t fit all, thus highlighting the importance of understanding which training method is better suited for each surgeon. Our corpse model with hemodynamic simulation for VATS lobectomy training could be suggested both for residents and surgeons who have already begun learning, but who want to improve some specific steps of the procedure. Lastly, highly skilled surgeons may also appreciate our model, albeit in situations more focused on highly complex resections, such as sleeves or segmentectomies.

To wrap up, we have proposed an innovative beating-heart and filled-vessel cadaveric model for the simulation of VATS lobectomies.

From our preliminary experience, the model is cost effective, smooth to develop, and realistic for thoracoscopy surgical simulation, especially for younger surgeons.

## Supplementary Information

Below is the link to the electronic supplementary material.

Supplementary file1 (MP4 12626 KB)

Supplementary file2 (MP4 74746 KB)
